# Colonic Biopsies to Assess the Neuropathology of Parkinson's Disease and Its Relationship with Symptoms

**DOI:** 10.1371/journal.pone.0012728

**Published:** 2010-09-14

**Authors:** Thibaud Lebouvier, Michel Neunlist, Stanislas Bruley des Varannes, Emmanuel Coron, Anne Drouard, Jean-Michel N'Guyen, Tanguy Chaumette, Maddalena Tasselli, Sébastien Paillusson, Mathurin Flamand, Jean-Paul Galmiche, Philippe Damier, Pascal Derkinderen

**Affiliations:** 1 UMR 913, Inserm, Nantes, France; 2 CIC-04, Inserm, Nantes, France; 3 UFR Médecine, Université de Nantes, Nantes, France; 4 UFR Sciences et Techniques, Université de Nantes, Nantes, France; 5 Service de Neurologie, CHU Nantes, Nantes, France; 6 Institut des Maladies de l'Appareil Digestif (IMAD), CHU Nantes, Nantes, France; 7 Pôle d'Information Médicale, Évaluation et Santé Publique (PIMESP), CHU Nantes, Nantes, France; National Institutes of Health, United States of America

## Abstract

**Background:**

The presence of Lewy bodies and Lewy neurites (LN) has been demonstrated in the enteric nervous system (ENS) of Parkinson's disease (PD) patients. The aims of the present research were to use routine colonoscopy biopsies (1) to analyze, in depth, enteric pathology throughout the colonic submucosal plexus (SMP), and (2) to correlate the pathological burden with neurological and gastrointestinal (GI) symptoms.

**Methodology/Principal Findings:**

A total of 10 control and 29 PD patients divided into 3 groups according to disease duration were included. PD and GI symptoms were assessed using the Unified Parkinson's Disease Rating Scale part III and the Rome III questionnaire, respectively. Four biopsies were taken from the ascending and descending colon during the course of a total colonoscopy. Immunohistochemical analysis was performed using antibodies against phosphorylated alpha-synuclein, neurofilaments NF 220 kDa (NF) and tyrosine hydroxylase (TH). The density of LN, labeled by anti-phosphorylated alpha-synuclein antibodies, was evaluated using a quantitative rating score. Lewy pathology was apparent in the colonic biopsies from 21 patients and in none of the controls. A decreased number of NF-immunoreactive neurons per ganglion was observed in the SMP of PD patients compared to controls. The amount of LN in the ENS was inversely correlated with neuronal count and positively correlated with levodopa-unresponsive features and constipation.

**Conclusion/Significance:**

Analysis of the ENS by routine colonoscopy biopsies is a useful tool for pre-mortem neuropathological diagnosis of PD, and also provides insight into the progression of motor and non-motor symptoms.

## Introduction

Normal function of the gastrointestinal (GI) tract relies both on intrinsic reflexes and extrinsic control. The extrinsic innervation depends on parasympathetic and sympathetic outputs. The intrinsic innervation relies on the enteric nervous system (ENS), an integrative neuronal network organized in two main plexuses, myenteric and submucosal, that control bowel motility and transmucosal fluid exchange, respectively [1]. A wide range of GI diseases associated with motility dysfunction can be considered, in part, as extrinsic and/or enteric neuropathies [2]. An emerging concept is that the field of enteric neuropathies extends well beyond digestive diseases, and that a subset of central nervous system (CNS) disorders may present with concomitant alterations of the ENS [3], [4], [5]. Among those, Parkinson's disease (PD) is likely to be a prime example because alterations of the ENS and GI dysfunction have been described in the course of the disease [6]. Whether these alterations mirror brain pathology, and how they relate to clinical symptoms, remain open questions.

PD is indeed much more than a selective degeneration of the substantia nigra. The loss of nigral dopaminergic neurons is responsible for the cardinal motor symptoms of PD (i.e. bradykinesia and/or rest tremor), that are improved by dopamine replacement therapy [7]. Yet PD patients also suffer from a wide variety of dopa-unresponsive symptoms likely to reflect lesions beyond the substantia nigra [8]. Most of the non-dopaminergic symptoms appear or worsen with advancing age and disease progression, and represent the majority of the disability observed in advancing PD [9]. They include dysautonomia and axial symptoms, such as dysarthria, gait and postural instability, and cognitive decline [10]. Among GI symptoms, chronic constipation (CC) is by far the most frequent, affecting up to 60% of PD patients [11].

The pathological hallmarks of PD are neuronal inclusions termed Lewy bodies and Lewy neurites (LN) whose main component is aggregated and phosphorylated alpha-synuclein [12], [13], [14]. PD pathology concentrates in susceptible regions of the CNS and peripheral autonomic nervous system, including the ENS [15]. Lewy bodies within the ENS, first reported in 1984 [16], provide a putative anatomical basis for GI symptoms [17].

We have recently shown that whole-mounts of submucosa from routine colonic biopsies allow a morphological analysis of the submucosal plexus (SMP) [18], [19]. Using this technique in a pilot study, we have demonstrated that 4 out of 5 PD patients display Lewy pathology. Nevertheless, the small number of patients included did not enable us to draw any clinicopathological correlations or to assess the pathology in detail. We have therefore conducted the present study in a larger set of 30 PD patients to allow in depth analysis of enteric pathology throughout the colonic SMP, and to correlate the extent of pathology with motor symptoms and constipation.

## Methods

### Subjects

PD patients aged 40–75 years were recruited over 24 months from the movement disorder clinic in Nantes University Hospital, France. Diagnosis was made according to the United Kingdom Parkinson's Disease Survey Brain Bank [20]. To limit recruitment bias and in order to span the entire course of PD, 3 groups of patients divided according to disease duration were included (group 1: ≤6 years, group 2: 7–12 years and group 3: ≥13 years disease duration).

Healthy patients requiring a total colonoscopy for colorectal cancer screening were included as controls. None of the control subjects had a history of neurological or psychiatric diseases.

### Patient evaluation

In PD patients, motor symptoms were assessed using the Unified Parkinson's Disease Rating Scale part III (UPDRS-III) [21]. UPDRS-III was performed only in ON-state for group 1 and in both OFF and ON-state for groups 2 and 3. OFF-state was obtained following an overnight withdrawal of dopaminergic treatment, and ON-state was reached one hour after intake of the normal morning dose. Dopa-responsiveness was defined as the percentage of UPDRS-III improvement compared with baseline. UPDRS-III score was subdivided into an axial score (sum of items 18, 19, 22 and 27–30) that evaluates symptoms such as dysarthria or postural instability [22].

Assessment of GI symptoms was performed using the Rome III questionnaire. Chronic functional constipation was diagnosed as defined by Rome III criteria [23]. The sum of the 6 constipation items on the Rome III questionnaire (questions 9 to 14) was used as a semi-quantitative score to assess the severity of CC.

All controls underwent a neurological examination to rule out PD symptoms and cognitive deficiency. The study protocol was approved by the local Committee on Ethics and Human Research (Comité de Protection des Personnes Ouest VI), and registered on ClinicalTrials.gov (identifier NCT00491062). Written informed consent was obtained from each patient and from each normal volunteer.

### Colonoscopy biopsies

A total colonoscopy was performed according to the usual procedure of the Gastroenterology department of Nantes University Hospital. In both patients and controls, 4 biopsies were taken in the ascending colon and descending colon, respectively. Biopsies were performed using standard biopsy forceps without needles (FB210K, Olympus co., Japan). Samples were immediately immersed in 4°C saline solution and processed as described.

### Immunohistochemistry

Submucosa samples were processed for whole-mount immunostaining as described previously [18]. The primary antibodies used were those directed against phosphorylated alpha-synuclein (1∶5000, WAKO, Osaka, Japan), neurofilament H 200 kDa (NF, 1∶250, Chemicon, USA), Hu C/D (1∶200, Invitrogen, Cergy Pontoise, France), tyrosine hydroxylase (TH, 1∶500, Pel-Freez, USA) and dopamine-beta-hydroxylase (DBH, 1∶250, Millipore, USA). Suitable secondary antibodies conjugated to Alexa Fluor 488, 594 and 647 were used (Invitrogen, Cergy-Pontoise, France).

### Neuronal cell counting and scoring

Neuronal counts were performed in one submucosa sample from the ascending and descending colon, respectively. Hu or NF-immunoreactive (IR) neurons were counted in all available ganglia of the sample using a Zeiss Axiovert 200 M (Zeiss, Thornwood, NY). The results were expressed as the average of the mean number of neurons per ganglion in the two biopsies.

Density of phosphorylated alpha-synuclein inclusions was evaluated after analyzing 2 biopsies from ascending and 2 from descending colon. A biopsy was considered positive when containing at least 1 LN. During the study, we used alternatively a 3-category *semi-quantitative* scale based on the subjective assessment of LN density in all 4 biopsies considered as a single sample, and a *quantitative* rating scale based on the proportion of positive biopsies (0: *absent*; +: 1/4 positive biopsy: *moderate*; ++: ≥2/4 positive biopsies, *severe* pathology). As the two methods yielded similar results (4/29 mismatches), we chose to present only the quantitative rating scale because of its higher reproducibility.

### Statistical analysis

Data are presented as mean ± standard deviation. For graphical representation of the total population of patients (n = 29) and controls (n = 10), box plots were used in which the end of the whiskers represent the minimum and maximum scores, and ‘+’ sign represents the mean.

Regarding the number of neurons per ganglion, differences between patients and controls were analyzed by unpaired two-tailed Student's t-tests. Differences between subgroups were analyzed by two-way ANOVA followed by post hoc Newman–Keuls tests.

For ordinal data (clinical scores), conventional Mann-Whitney and Kruskal-Wallis tests followed by post hoc Dunn's analyses served to compare median magnitudes of change.

Correlation between Lewy pathology score and other parameters were assessed by Spearman test. Adjustment with age was done with multiple linear regression. Chi square tests were used for frequency analysis. For all statistical tests p<0.05 was deemed significant.

## Results

A total of 30 PD patients and 10 controls were recruited. Of these 30 patients, one was excluded because of an error in the processing of their biopsy. Patients were subdivided into groups based on disease progression, resulting in similar group sizes (9 in group 1, ≤6 years; 10 in group 2, 7–12 years; 10 in group 3, ≥13 years disease duration). ***Table 1*** shows the main clinical features and pathological scores of all patients. Age and sex did not differ significantly between patients and controls. CC, as defined by Rome III criteria, affected one of the controls (10%) and 23 out of 29 PD patients (79%, p<0.001) (***table 2***).

**Table 1:** Main clinical characteristics and immunohistochemical findings in patients.

Patient	Group	Sex	Age	Disease duration (years)	UPDRS-III axial subscore	Dopa-responsiveness	Chronic functional constipation Y/N	Sum of Rome III constipation items	Neurons per ganglion	Lewy pathology quantitative score
**1**	1	F	44	1	2	–	Y	2	4.4	0
**2**	1	M	67	2	11	–	Y	3	4.0	++
**3**	1	F	72	2	10	–	Y	3	2.6	++
**4**	1	M	47	4	4	–	N	0	3.5	+
**5**	1	M	66	4	1	–	Y	3	3.7	+
**6**	1	F	58	5	8	–	Y	4	4.4	++
**7**	1	F	56	5	3	–	N	1	3.7	0
**8**	1	M	58	6	5	–	Y	3	4.8	+
**9**	1	M	71	6	17	–	N	0	3.7	++
**10**	2	M	63	8	5	70%	N	1	4.0	0
**11**	2	M	55	9	3	57%	Y	2	3.5	+
**12**	2	M	63	9	4	72%	Y	4	3.1	+
**13**	2	F	64	9	5	*md* *	Y	2	4.4	0
**14**	2	M	66	9	4	77%	Y	4	5.9	0
**15**	2	M	63	10	1	61%	Y	3	3.8	+
**16**	2	F	65	10	5	41%	Y	4	4.6	+
**17**	2	M	69	10	9	100%	N	3	3.2	0
**18**	2	F	48	12	1	93%	N	0	4.5	0
**19**	2	M	65	12	7	44%	Y	3	2.6	++
**20**	3	M	64	13	5	50%	Y	4	2.6	+
**21**	3	F	66	13	9	78%	Y	4	3.8	0
**22**	3	F	69	13	10	41%	Y	3	3.4	++
**23**	3	F	57	14	4	79%	Y	3	3.9	+
**24**	3	F	68	14	1	72%	Y	3	4.5	+
**25**	3	M	65	16	4	72%	Y	6	2.5	++
**26**	3	M	68	19	4	74%	Y	4	2.6	++
**27**	3	F	71	20	4	86%	Y	2	3.6	+
**28**	3	M	72	20	21	29%	Y	4	3.5	++
**29**	3	M	60	24	13	57%	Y	5	2.4	++

*md: missing data.

**Table 2:** Comparison of main clinical and immunohistochemical variables between patients and controls.

Parameters	Controls mean ± SD	Parkinson's mean ± SD	*p*-value
**Age**	58.6±7.2	62.8±7.4	0.131
**Gender (% male)**	58.6%	60.0%	1.000
**Chronic constipation**	10%	79%	0.0002***
**Neurons per ganglion**	4.3±0.3	3.7±0.2	0.040*
**Lewy neurites (% positive patients)**	0%	72%	0.0001***

### Lewy neurites in colonic biopsies from PD patients

**Figure 1:**
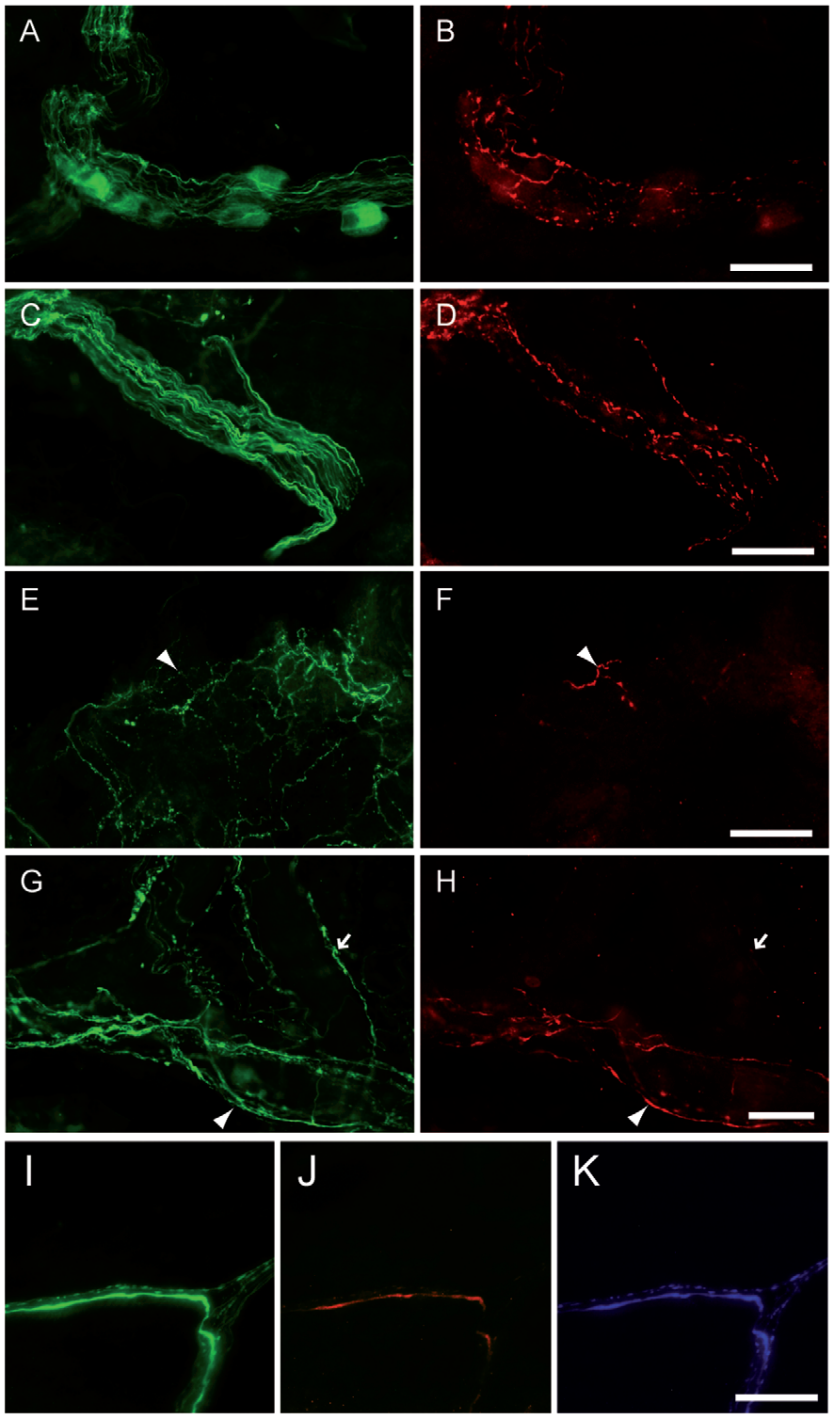
Phospho-α-synuclein-positive submucosal neurites in PD patients. Labeling with antibodies against neurofilament (NF) (**ACEGI**) and phosphorylated α-synuclein (**BDFHJ**) revealed that some NF-immunoreactive (IR) neurites were also phospho-α-synuclein-IR. Occasionally, these phospho-α-synuclein-IR neurites were present amidst a submucosal ganglion (**AB**). Some of these structures formed bundles (**D**) while others were isolated (**arrowhead in F**). Thirty seven percents of the phospho-α-synuclein-IR neurites were perivascular (**GH**). Triple immunostaining with antibodies against tyrosine hydroxylase (**K**) revealed that 60% of LN were also TH-immunoreactive (**IJK**). Scale bar: 30 µm.

Twenty-one out of 29 PD patients (72%) displayed Lewy pathology, in the form of Lewy neurites (LN) immunoractive (IR) for both neurofilament (NF) and phosphorylated alpha-synuclein (***figure 1A–D***, ***table 2***). No immunoreactivity for phosphorylated alpha-synuclein was observed within enteric neurons in controls, with the exception of some faint somatic labeling that was present in both patients and controls (data not shown). The proportion of patients with Lewy pathology did not correlate with disease progression (78% positive in group 1, 50% positive in group 2 and 90% positive in group 3).

LN were observed in isolated or bundled fibers (***figure 1C–F***). Triple immunostaining experiments showed that 60% of the LN were also IR for tyrosine hydroxylase (TH). Thirty-seven percent of the LN were perivascular (***figure 1GH***), and 92% perivascular LN were TH-IR (***figure 1I–K***). Additional experiments performed in a subset of 6 positive PD patients (patients 16, 19, 22, 26, 28 and 29) showed that 51% of LN also expressed DBH (***figure S1***). No cytoplasmic Lewy body labeling was observed. 72% of patients exhibited phosphorylated alpha-synuclein-positive labeling (PS+ patients). However the pathological burden was strikingly disparate between PS+ patients: some displayed abundant LN in most samples, while others displayed only one positive inclusion in a single biopsy. Postulating that the density of LN was a more relevant marker than their mere presence/absence, we used a quantitative Lewy pathology score. Group 0 represented the negative cases (n = 8) while groups + (n = 11) and ++ (n = 10) represented moderate and severe Lewy pathology, respectively.

### Neurofilament and tyrosine hydroxylase-expressing neurons in the submucosal plexus

**Figure 2:**
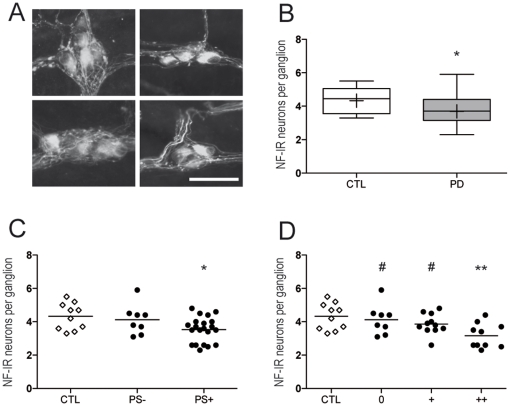
Count of neurofilament-positive neurons in the submucosal plexus of PD patients. **A.** Neurofilament-immunoreactive (NF-IR) submucosal neurons were counted in every available ganglion from colonic biopsies. Representative photographs of ganglia from PD patients (left panels) and controls (right panels). Scale bar: 30 µm. **B.** A significant decrease in the number of NF-IR neurons per ganglion was present in the SMP of PD patients (PD, n = 29) as compared to controls (CTL, n = 10) (p<0.05). The bottom and the top of the box represent the 25th and 75th percentiles, respectively, and the end of the whiskers represent the minimum and maximum values; the median is represented as a bar and the mean as a ‘+’ sign inside the box. **C.** When segregating patients according to the presence (PS+) or absence (PS-) of phospho-synuclein IR neurites, the difference between patients and controls was sustained only for the group with Lewy pathology (PS+, n = 21, p<0.05). **D.** When further stratifying patients according to the density of pathology, the difference between patients and controls was sustained only for the group with severe Lewy pathology (++, n = 10, p<0.01). Groups without (0) or with moderate pathology (+) significantly differed from the group with severe (++) pathology (p<0.05). Each white square represents one control, each black circle represents one PD patient. Horizontal bars represent the mean. *p<0.05 and **p<0.01 as compared with controls. #p<0.05 as compared with the group with severe pathology (++).

In order to assess the suitability of NF as a neuronal marker in human SMP, we first performed a double Hu and NF-immunostaning in a subset of 3 control and 3 PD patients (patients 9, 17 and 27). Anti-NF 200 kDa antibody virtually labels all submucosal neurons in both conditions (***figure S2***), thus allowing the use of NF-immunostaining for neuronal count. Control submucosal samples displayed 4.3±0.8 NF-IR neurons per ganglion. In PD, there was a decreased number of NF-IR neurons per ganglion (3.7±0.8; p = 0.04) (***figure 2AB***, ***table 2***).

When patients were separated into two groups according to the presence (PS+) or absence (PS-) of phospho-synuclein IR neurites, only PS+ patients had a significant drop in the amount of NF-IR when compared to controls (p = 0.01) (***figure 2C***). When PS+ patients were further stratified into subgroups with moderate (+) and severe (++) pathology, there was a highly significant difference in NF-IR between PS+ patients with severe pathology (++) and the control group (p<0.01), and furthermore significant differences were apparent between the severe pathology group and those with absent (0) and moderate (+) pathology (p<0.05) (***figure 2D***). After adjustment for age, a significant correlation remained between Lewy score and the number of NF-IR neurons per ganglion (p = 0.02). There was no correlation between the number of neurons per ganglion and age (p = 0.193), nor between the number of neurons per ganglion and disease duration (p = 0.094), including after age-adjustment (p = 0.479).

### Clinicopathological correlations

We then sought to correlate our two primary histological findings, namely LN and the number of NF-IR neurons, with both neurological and chronic constipation (CC) symptoms (***table 3***).

**Table 3:** Clinico-pathological correlations.

Correlations	Age	Disease duration (years)	UPDRS-III axial subscore	Dopa-responsiveness	Sum of Rome III constipation items	Neurons per ganglion	Lewy pathology quantitative score
**Spearman's correlation with quantitative score (p values)**	0.034 *	0.290	0.008 **	0.007 **	0.042 *	0.004 **	0
**Age-adjusted correlation with quantitative score (p values)**		0.507	0.004 **	0.006 **	0.17	0.02 *	0

#### a. Neurological

**Figure 3:**
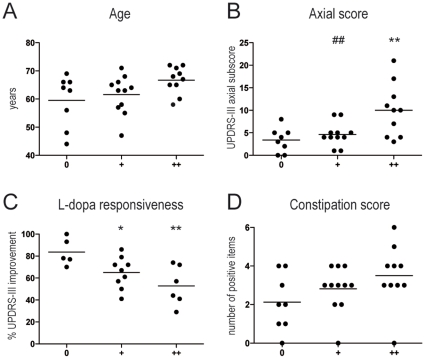
Correlation of clinical symptoms with pathology burden. **A.** Measure of pathology burden using a quantitative score correlated with age, which appeared as a potential confounding factor. Subsequent correlation analysis was performed after adjusting the data for age. **B.** Pathology burden positively correlated with axial score, which measures axial symptoms such as dysarthria and postural instability. The group with severe pathology (++) significantly differed from the group with absent (0) or moderate (+) pathology (p<0.01). **C.** Pathology burden also correlated with L-Dopa responsiveness, estimated by the percentage of UPDRS-III improvement after L-Dopa intake. Responsiveness was higher in the group with absent pathology (0), as compared with the group with moderate (+, p<0.05) or severe (++, p<0.01) pathology. **D.** Pathology burden also correlated with constipation severity, as defined by the number of positive answers to the constipation items of Rome III questionnaire. Each black circle represents one PD patient. Horizontal bars represent the mean. *p<0.05 and **p<0.01 as compared with the group with absent pathology (0). #p<0.05 and ##p<0.01 as compared with the group with severe pathology (++).

In order to correlate Lewy pathology with clinical features, we stratified patients according to the quantitative Lewy pathology score, as described above. The Lewy pathology score positively correlated with age (r_S_ = 0.395; p = 0.03) (***figure 3A***). Age is associated with worsening prognosis of PD [24], and as such is a potential confounding factor. Therefore, all subsequent correlations between Lewy pathology and disease severity were performed after adjusting for age.

There was no correlation between pathology and disease duration. Axial score was higher in the group with severe Lewy pathology (++) when compared to the groups with absent (0) or moderate (+) pathology (p<0.01) (***figure 3B***), and axial score positively correlated with the Lewy pathology score (p = 0.004). Further reinforcing these findings, dopa-responsiveness negatively correlated with the severity of pathological burden (p = 0.0064). The group devoid of LN was significantly more responsive to levodopa than the groups with either moderate (p<0.05) or severe (p<0.01) pathology (***figure 3C***). Conversely, the number of neurons per ganglion did not correlate with either axial score or dopa-responsiveness.

#### b. GastrointestinaI

CC was significantly more frequent among PS+ (19/21) than among PS- patients (4/8) (p<0.05, Fisher's exact test). The severity of CC, as assessed by the constipation score, positively correlated with the Lewy pathology score (r_S_ = 0.381; p = 0.042) (***figure 3D***). However, this correlation was not significant after adjusting for age (p = 0.17). There was no correlation between the number of neurons per ganglion and CC score (p = 0.56).

## Discussion

The four main outcomes of the present survey are (1) the demonstration of LN in the SMP of 72% of PD patients, but in none of the controls, (2) a higher frequency of constipation in LN-positive patients, (3) a strong correlation between LN burden and disease severity, (4) the possibility to readily and reproducibly analyze the ENS in living patients, thereby providing an opportunity to develop an original biomarker for PD.

### Lewy pathology in the SMP of PD patients

A major finding of our study was the identification and characterization of neuropathological lesions in the colonic submucosa of PD patients. Lewy pathology in the SMP was composed of LN only. This is consistent with a recent report in which alpha-synuclein inclusions observed in the gastric submucosa of deceased PD patients were LN, while Lewy bodies were present in the soma of myenteric neurons [25]. The absence of Lewy bodies in the SMP precludes affirmation that the SMP is intrinsically affected by the pathological process. Indeed, Lewy bodies and LN have been reported in colonic and gastric myenteric neurons of deceased PD patients ([25] and our unpublished results). In the guinea pig, myenteric neurons have been shown to project to the SMP as well as to the submucosal blood vessels [26].

Intrinsic dopaminergic neurons in the myenteric plexus are altered in PD both in human [27] and animal models [28]. However, intrinsic dopaminergic neurons are a minority in the colonic SMP [29]. At the level of the colonic SMP, the majority of TH-IR neurites are noradrenergic sympathetic axons that also express DBH (94% in our unpublished data). Our present results show that 37% and 60% of LN are perivascular and TH-IR respectively, and that 51% are DBH-IR, suggesting that many TH-IR LN belong to postganglionic sympathetic neurons. Thus, a significant proportion of the enteric pathology reflects the widespread sympathetic degeneration seen in other systems [30], [31].

LN in the SMP was present in 21 out of 29 PD patients. The heterogeneity of PD with regard to peripheral autonomic alterations was underscored by two recent autopsy-based studies that found Lewy inclusions in the GI tract as well as in the sympathetic network of nearly three-quarters of PD patients [30], [32]. Whether this heterogeneity reflects different PD subtypes or disease severity will be further discussed.

Because of samples shortage, NF-co-immunostaining was intended both for localizing phosphorylated alpha-synuclein immunoreactivity within neurons and for neuronal counting, taking advantage of NF somatic labeling. A significant decrease in the number of NF-IR neurons was observed in the SMP of PD patients. The significance of this finding is debatable since the suitability of NF as an enteric neuronal marker has been challenged in a recent study showing that as low as 43% of Hu-IR neurons co-expressed NF in the myenteric plexus [33]. However in our experience, virtually all Hu-IR submucosal neurons display somatic NF immunoreactivity (***figure S2***). This discrepancy might result from differences in the expression of NF between the submucosal and myenteric plexus and/or from differences in the sensitivity of the antibodies that were used.

Although we cannot rule out phenotypic changes resulting in a decreased expression of NF in a subset of neurons, it is tempting to attribute the drop in NF-IR to neuronal loss. Whether enteric neuron loss occurs in PD is still unclear. Two earlier studies performed on biopsies and surgical samples failed to show any neuronal loss in the colonic SMP of PD patients [19], [27]. The low number of patients assessed in these studies probably accounts for this discrepancy. If enteric neurodegeneration occurs in the course of PD, the cardinal neuropathology of the disease, namely neuronal death and Lewy inclusions, would be recapitulated in the ENS, thereby mirroring the lesions of affected brainstem nuclei in the CNS.

### Lewy pathology burden and constipation

GI dysfunction stands among the most common non-motor symptoms of PD. Symptoms such as dysphagia, nausea, gastroparesis, and bowel dysfunction, including both reduced bowel movement frequency and dyschesia, are a significant cause of disability [34]. CC was significantly more frequent in the group with than without LN, suggesting a pathogenic role for inclusions.

In this aspect however our study suffers from two potential limitations. First, we did not use a validated constipation severity score. Scores such as Patient Assessment of Constipation Symptoms (PAC-SYM) questionnaire [35] might have revealed stronger correlations between pathology burden and CC, while the correlation we found did not remain significant after adjustment for age. Second we did not assess the MP, which is directly involved in the control of bowel motility. Whether the density of LN in the SMP is representative of the pathology burden in the MP is still an open question that could be addressed by a comparative and comprehensive analysis of the myenteric and SMP in surgical or postmortem specimens.

Although controversial, CC has been linked to increased age-related neurodegeneration in the ENS [36]. Loss of submucosal neurons [37], [38] and alterations of the sympathetic innervation [39] in the ageing rat have been implicated in the pathophysiology of CC, and a loss of myenteric neurons has been demonstrated in the colon of patients with CC [40]. From our study, CC in PD does not appear to be related with the number of submucosal NF-IR neurons, and degeneration of sympathetic innervation might play a role in this feature, since a significant proportion of LN belonged to sympathetic outputs.

### Lewy pathology burden is correlated with PD progression

All patients included in this study had a comprehensive neurological assessment. This enabled us to draw parallels between pathological burden in the ENS and Parkinsonian symptoms.

The density of submucosal LN was significantly correlated with the presence of dopa-unresponsive axial symptoms, such as dysarthria or postural instability but not with disease duration and motor symptoms. Two recent studies relating autonomic dysfunction with the clinical phenotype of PD provided similar results [41], [42]. Clinical scores of autonomic symptoms, postural blood pressure response impairment [41] and myocardial ^123^I-metaiodobenzylguanidine uptake [42] weakly correlated with disease duration and motor symptom severity. Conversely, the presence of axial symptoms was associated with greater autonomic dysfunction. These studies, together with our results, strongly suggest that functional and structural alterations in the enteric and autonomic nervous systems are associated with the presence of axial motor symptoms. Interestingly, a recent survey searching for patterns of coherency among the full clinical spectrum of PD found that dysautonomic, axial and cognitive symptoms co-segregated and best characterized disease severity [43]. In an individual patient, the appearance of axial symptoms is predictive of disease progression toward dementia [10] and is thought to reflect the spreading of pathology to non-dopaminergic structures of the brainstem, forebrain and cortex [44]. Thus, the heterogeneity of PD regarding dysautonomia in general, and alterations of the ENS in particular, might reflect in part different degrees of severity.

### The ENS as biomarker in PD

Routine colonic biopsies can be used to provide examination of the submucosal enteric neurons in living patients [45]. Here we confirm on a large scale that such a procedure allows a safe and reliable analysis of the ENS. Total colonoscopy is a simple diagnostic procedure with a low risk of adverse effects [46]. Accordingly, no complications occurred in the 40 patients included in the present study, either during or after the procedure.

The skin and the olfactory epithelium contain neuronal networks affected by Lewy pathology during PD that are also accessible by routine biopsies and have recently been evaluated as histopathological markers for PD [30], [47]. However, the results of these works were disappointing since only 2 out of 20 PD patients displayed LN in skin biopsies and no alpha-synuclein aggregates were present in the biopsied olfactory epithelium of 7 Parkinsonian patients [48], [49]. Consequently, our study is the first to show that Lewy pathology can be reproducibly analyzed using biopsies from a peripheral tissue in living patients.

The ENS displays specific features that make it a prime candidate for being a histopathological marker of PD (for review see [50]). In contrast to the skin and olfactory epithelium, colonic biopsies allow the retrieval and analysis of a dense integrated neuronal network, not only neuronal processes [18]. Using optical recording techniques, electrophysiological properties of submucosal neurons from colonic biopsies can be studied [51]. Therefore, analysis of the ENS during the progression of PD may represent a unique opportunity to monitor PD pathology and its impact on neuronal function in living patients. We have shown in the present survey that the pathological burden in the ENS is correlated to the presence of dopa-unresponsive axial symptoms, strongly supporting the use of colonic biopsies as a biomarker for the assessment of PD severity. Their use for the positive diagnosis of incipient or even preclinical PD still requires to test the specificity of enteric submucosal LN in larger series and to improve the sensitivity of the technique. Possible strategies include an increased number of colonic samples or the use of upper digestive tract biopsies, which add the potential risk of inhalation during the endoscopy.

Braak and coworkers have postulated that the ENS is affected early by Lewy pathology during the course of PD, even before the pathology is apparent in the substantia nigra. This suggests that the ENS heralds the onset of a pathological process that further spreads to the CNS via autonomic innervation of the gut [25], [52]. Although tempting, this theory relies only on correlations performed in autopsy studies and is still a matter of debate. By demonstrating the presence of LN in the colon at early stages (78% of patients <6 years), our findings do not refute this hypothesis. We believe that the use of colonic biopsies, by enabling analysis of the ENS in PD patients at a very early stage of the disease, will be helpful for validating or refuting Braak's hypothesis.

In conclusion, the ENS can be considered not only as ‘the second brain’ [53], but also as a window towards the ‘first’ brain. The ENS probably antedates the CNS in evolutionary terms, and its complexity challenges its central counterpart, especially since the functional and chemical diversity of enteric neurons closely resembles that of the CNS [54]. Enteric neuropathies recapitulate many aspects of neurological diseases [2]. In particular, degenerative changes occur in the aging gut [55]. In this context, it is hardly surprising that enteric neurons can mirror central alterations in neurodegenerative disorders. It is possible that further studies may expand this concept to other neurodegenerative diseases. For example, the presence of hyperphosphorylated tau aggregates in myenteric neurons of aging rats suggests that tauopathies such as Alzheimer's disease may also affect the ENS [56]. We consider our method to represent a major advance in the search for biomarkers for PD. The use of the, as yet unrecognized, ENS as a window into the CNS represents an original approach, with implications that may well extend beyond PD.

## Supporting Information

Figure S1 51% of phospho-α-synuclein-positive submucosal neurites express DBH. Labeling with antibodies against dopamine-beta-hydroxylase (DBH) (BDF) and phosphorylated α-synuclein (ACE) revealed that some DBH-immunoreactive (IR) neurites were also phospho-α-synuclein-IR. In a subset of 6 PD patients, the proportion of Lewy neurites that expressed DBH was 51%. Perivascular Lewy neurites in EF. Scale bar 30 µm.
(3.90 MB PDF)

Figure S2 Evaluation of neurofilament immunostaining as a pan-neuronal marker in human submucosal plexus. Labeling with antibodies against neurofilament 200 kDa (NF) (ACEGIK) and Hu C/D (BDFHJL) revealed that virtually all submucosal neurons, whether isolated (EF and KL) or in submucosal ganglia containing >2 neurons, coexpress NF and Hu C/D. Sample images from 3 controls (A–F) and 3 PD patients (G–L). Note the nuclear expression of Hu in L, a pattern that is occasionally seen in patients and controls. Scale bar 30 µm.
(8.13 MB PDF)
