# Supplementary figures and images for: Colonic Biopsies to Assess the Neuropathology of Parkinson's Disease and Its Relationship with Symptoms
Source: PLoS One. 2010 Sep 14;5(9):e12728. doi: 10.1371/journal.pone.0012728 (PMC2939055; doi:10.1371/journal.pone.0012728)

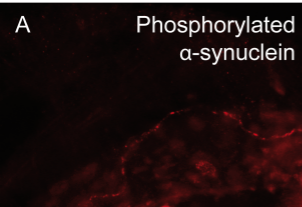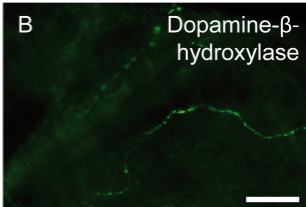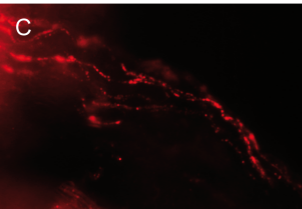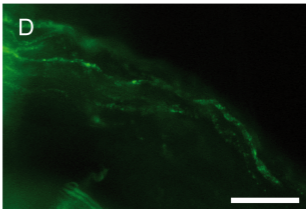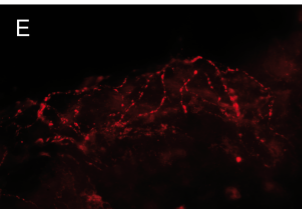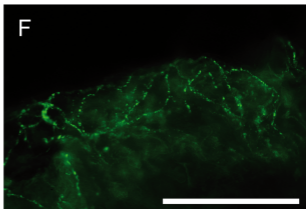

Supplement: Figure S1 — 51% of phospho-α-synuclein-positive submucosal neurites express DBH. Labeling with antibodies against dopamine-beta-hydroxylase (DBH) (BDF) and phosphorylated α-synuclein (ACE) revealed that some DBH-immunoreactive (IR) neurites were also phospho-α-synuclein-IR. In a subset of 6 PD patients, the proportion of Lewy neurites that expressed DBH was 51%. Perivascular Lewy neurites in EF. Scale bar 30 µm. (3.90 MB PDF) [file pone.0012728.s001.pdf]

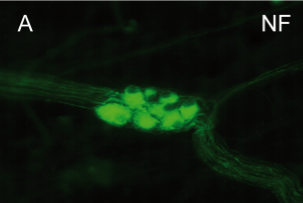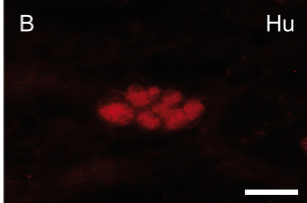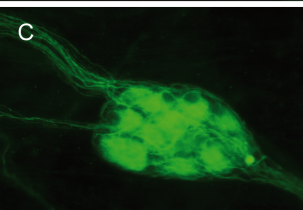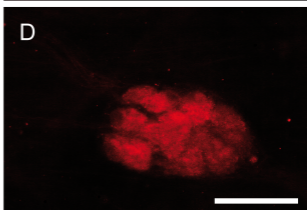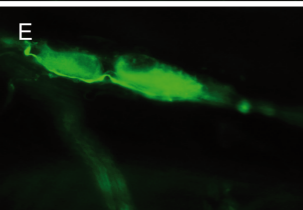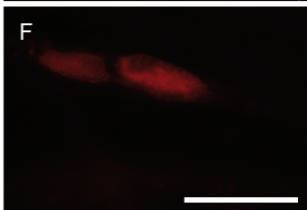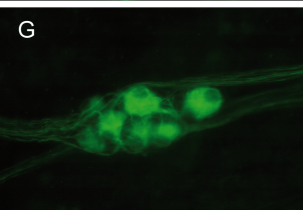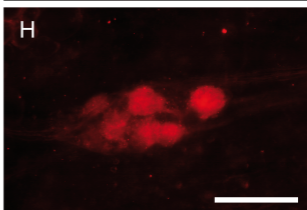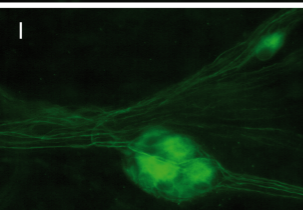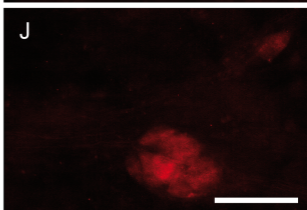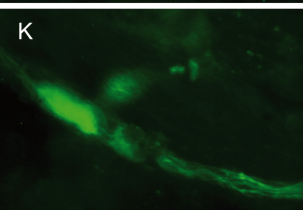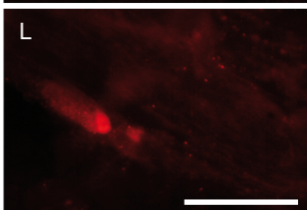

Supplement: Figure S2 — Evaluation of neurofilament immunostaining as a pan-neuronal marker in human submucosal plexus. Labeling with antibodies against neurofilament 200 kDa (NF) (ACEGIK) and Hu C/D (BDFHJL) revealed that virtually all submucosal neurons, whether isolated (EF and KL) or in submucosal ganglia containing >2 neurons, coexpress NF and Hu C/D. Sample images from 3 controls (A–F) and 3 PD patients (G–L). Note the nuclear expression of Hu in L, a pattern that is occasionally seen in patients and controls. Scale bar 30 µm. (8.13 MB PDF) [file pone.0012728.s002.pdf]
